# Wearable IMU for Shoulder Injury Prevention in Overhead Sports

**DOI:** 10.3390/s16111847

**Published:** 2016-11-03

**Authors:** Samir A. Rawashdeh, Derek A. Rafeldt, Timothy L. Uhl

**Affiliations:** 1 Electrical and Computer Engineering, University of Michigan, Dearborn, MI 48128, USA; 2 Athletic Training and Rehabilitation Sciences, University of Kentucky, Lexington, KY 40536-0200, USA; Derek.rafeldt@uky.edu (D.A.R.); tluhl2@uky.edu (T.L.U.)

**Keywords:** wearable technology, inertial measurement unit, shoulder, injury prevention

## Abstract

Body-worn inertial sensors have enabled motion capture outside of the laboratory setting. In this work, an inertial measurement unit was attached to the upper arm to track and discriminate between shoulder motion gestures in order to help prevent shoulder over-use injuries in athletics through real-time preventative feedback. We present a detection and classification approach that can be used to count the number of times certain motion gestures occur. The application presented involves tracking baseball throws and volleyball serves, which are common overhead movements that can lead to shoulder and elbow overuse injuries. Eleven subjects are recruited to collect training, testing, and randomized validation data, which include throws, serves, and seven other exercises that serve as a large null class of similar movements, which is analogous to a realistic usage scenario and requires a robust estimator.

## 1. Introduction

In 2014, musculoskeletal disorders such as sprains, strains, and tears accounted for 420,870 nonfatal injuries requiring time off from work. Upper extremity injuries accounted for 346,170 injuries, of which 25% were shoulder injuries [1]. Musculoskeletal disorders (MSD) are well detailed in the literature as being due to awkward postures, repetitive activities, and high load demands [2]. Current practice to evaluate work postures and task demands come from observational or video analysis and self-reports. Observations are typically taken for a period of time and volumes are estimated over an 8 h work day. The assessment of postures and physical demands would be more accurate if the observations were performed over the entire day of performing a job [3]. Recently, researchers have been using microelectromechanical systems attached to the body to track motions. The sensors are yielding reasonably good comparisons to the gold standard of three-dimensional optical motion measures in the laboratory [4].These inertial measurement units (IMUs) are being used to track trunk and arm postures over the course of an entire day to determine the time spent in awkward trunk and arm postures for nurses [5] and dairy farmers [4]. These IMU devices provide an opportunity to record data without constant visual or video assessment [5].

Musculoskeletal disorders (MSD) caused by over-use in athletics are a major concern. In 2012, more than 1.35 million children under the age of 19 received medical treatment for sport and recreational injuries, a significant number of which were overuse injuries [6]. High school athletes sustain approximately 116,000 shoulder injuries yearly, of which 39% are musculoskeletal strains and sprains [7]. Due to the high risk of injury associated with overuse injuries, youth baseball has limited pitch counts during games to 75 [8,9]. However, the current evidence focuses on the number of pitches during game conditions, and this is not taking into account throwing during practice. Recently, spikes in training volume have been shown to increase the risk of injury [10]. Using the total workload of throws during a week of cricket, researchers examined the ratio of current week to the past 4 weeks of balls bowled and determined that a spike of 200% in workload increased the risk of injury 4 times [11]. Acute changes in physical activity have long been associated with overuse injuries [12]. The advent of body worn sensors such as IMUs now allows scientists and clinicians to monitor repetitions of motion better. The complexity of the throwing motion in multiple dimensions and using the upper extremity for other tasks such as running and batting creates a challenge. However, if an unsupervised monitoring system can capture throwing workloads, it would provide a better accounting of shoulder stresses in athletics, which can be used to issue preventative warnings.

We have developed a prototype wearable device to track arm motion using a set of inertial sensors. The device can be attached to an individual’s arm externally and record the acceleration (coupled measurements of the acceleration due to gravity, and that of the arm’s motion), rotation rates, and magnetic field direction for several hours. The data collected was used to develop a real-time approach for motion gesture recognition. The orientation of the arm as a function of time is estimated, and a classifier uses the raw measurements and derived orientation angle values to identify specific exercises and their counts from the data stream. The goal is to emulate a pedometer counting steps. However, the upper extremity involves more complex motion and performs many more functional tasks compared to the cyclic nature of walking/running, requiring a novel solution that can discriminate shoulder tasks and provide context. In a previous publication [13], we presented an overview and preliminary results of the algorithm developed. In this paper, we present a more detailed discussion, along with results from a validation dataset where subjects performed a wide range of exercises in a random order as a realistic challenge to the detection and classification algorithm.

## 2. Related Work

Traditionally, vision sensors have been used for motion capture and activity detection experiments [14,15]. This is often invasive as the subject must wear visual markers and confines the experiment to a laboratory setting or an environment with motion capture equipment. Using inertial sensors in a wearable unit enables non-intrusive activity monitoring through regular activities whether recreational or work. Previous medical applications of inertial gesture detection include Parkinson patients’ tremor measurement to evaluate the effect of medications [16], activity measurement of rheumatoid arthritis patients [17], and patient fall detection [18]. The previous work shows that a wearable inertial sensor node is a viable approach for our intended applications as a health monitor.

An extensive survey by Avci et al. of prior work on activity recognition using inertial sensing and potential applications in healthcare, sports, and wellbeing applications includes an outline of data segmentation approaches, feature extraction approaches, and classification techniques used in prior work [19]. Avci et al. observed that much of the prior work typically involves data collection and post-processing as separate steps and concludes that performing activity recognition in real-time “remains, therefore, an open research question”. The approach we are developing is intended to detect shoulder motion tasks in real-time in order to provide an active count of the shoulder activity history to help prevent injury or assess rehabilitation progress.

Gesture counting during sports involves processing large amounts of data in real-time, consisting of a multi-dimensional time series. When using an inertial measurement unit (IMU), there will be nine sensor values sampled at about 50–120 Hz (sensing acceleration, rotation rate, and magnetic field strength, each along three orthogonal axes). While classification techniques are well understood and capable of differentiating recorded actions (classifier accuracies range between 50% and 99% [19,20,21,22]), the problem is complicated by the windowing problem, which is rarely directly addressed. Specifically, automatically identifying the starting and ending points of a data sample to be classified is challenging, especially when searching for a heterogeneous set of actions where one threshold rule would not work for all motion gestures of interest. Windowing is often trivialized by having the user press a button to indicate the beginning or by assuming the gesture happens after a rest-pose and running the experiment in a controlled environment. For automatic windowing, prior work includes a discussion on segmenting one-dimensional time series into piece-wise linear segments [19,23]. However, an added constraint that does not appear in much of the prior work is the real-time requirement, which requires the development of a novel approach that does not require future knowledge of the signal. In essence, post-processing segmentation approaches that require the entire series a priori cannot be used in a real-time system.

## 3. Data Collection

**Figure 1:**
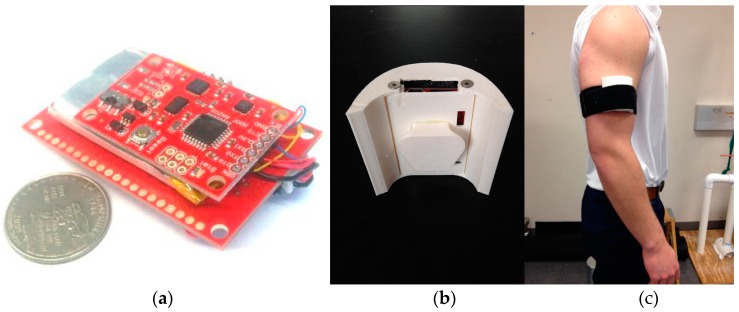
(**a**) Prototype IMU logger using off the shelf parts consisting of an IMU board on top, a data logger and power management board on bottom (facing away), and a lithium ion polymer battery between the two boards. Coin is a USA quarter; (**b**) The hardware was protected using a 3D-printed enclosure and (**c**) attached to each subject’s dominant arm as shown.

Hardware design: We developed a prototype modular IMU data logger shown in Figure 1. The unit consists of a sensor board that consists of triple-axis acceleration, angular rate, and magnetic field sensors. Specifically, the sensors used are the ITG-3200 MEMS triple-axis gyroscope (by InvenSense, San Jose, CA, USA), the ADXL345 triple-axis accelerometer (by Analog Devices, Norwood, MA, USA), and the HMC5883L triple-axis magnetometer (by Honeywell, Morris Plains, NJ, USA). The sensor board also contains a microcontroller that interfaces the sensors and performs basic data formatting to record the data. A data logging board records the sensor data onto a memory card and provides other functionality such as USB battery charging and file access. Data is logged at 50 Hz on the unit’s memory card. With our long-term goal of developing a wearable unit capable of sensing and processing the data on the local unit, we opted to develop our own prototype in favor of off-the-shelf data logging systems.

***Subjects.*** Eleven subjects with a mean age of 25 ± 7 years, mean height of 1.7 ± 0.1 m, and mean body mass of 77 ± 14 kg volunteered for this study. Potential subjects were recruited from a sample of convenience from a local college community. Subjects were excluded from this study if they reported a history of shoulder surgery or shoulder pain in the 6 months prior to participation. All subjects read and signed an informed consent form approved by the university’s institutional review board. The subjects were administered an Edinburgh Handedness Questionnaire to determine arm dominance and were then instrumented with the IMU data logger for testing [24].

**Figure 2:**
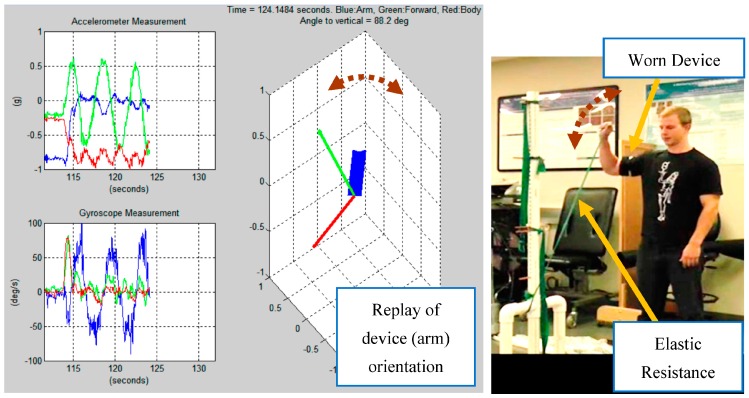
Data collection of an external rotation with arm elevated at 90°, a rehabilitation exercise. Tri-axis accelerometer and rate gyroscope plots are shown along with a playback of the unit’s orientation in space. Blue axis represents the length of the arm, green and red axes represent the remaining two orthogonal axes.

***Recorded movements.*** Data was collected using the prototype unit strapped onto the upper arm as shown in Figure 1. A wide range of exercises were collected to mimic the application scenario where the detection and recognition algorithm must deal with activities of daily living and a large null class, as discussed in the related work section. The exercises consisted of a series of seven shoulder rehabilitation exercises with elastic resistance and two sport activities as outlined in Table 1. The seven exercises were selected based on previous research that asserts that these resisted motions result in maximal overall shoulder complex musculature activation [25]. These exercises are likely to be performed as part of throwing warm-up by many overhead athletes. The two sport activities, throwing a baseball and hitting a volleyball, were chosen because they are similar in overhead functional demand but also generate different kinematic patterns [26,27,28]. Figure 2 shows a sample recording for an external rotation.

Each subject participated in a control data acquisition session followed by a randomized experimental data acquisition session with a five minute rest between sessions. The control data set was used for training and testing the algorithm and classifier. The randomized dataset was used for validation in which the co-authors that developed the algorithm were not aware of the specific motions that had been recorded. During the control data session, the subject performed each of the seven resisted exercises for eight repetitions at various compass headings to confirm that orientation based on earth’s magnetic field did not confound the measurements of the algorithm. The two sport activities were performed for eight repetitions as well. Baseball throwing and volleyball hitting were performed in a clinical laboratory using a sport net as a target and to allow the participants to simulate the functional tasks to the best of their ability without concern about accuracy. The order of elastic resistance exercises and sport activities and direction were exactly the same for all subjects during the control data collection portion of the study. The subjects were allowed five minutes to recover, and then the randomized data acquisition session was performed. The order of the elastic resistive exercises and the sport activities were randomized, the number of repetitions and compass direction was varied using a Latin Square table created prior to the beginning of data collection. The number of repetitions for each exercise was randomized using a random number generator between two and six. The number of baseball throws and volleyball hits was randomly generated between four and twelve in the same fashion. The order in which activities were performed and the number of repetitions for each activity was recorded on a data sheet. The co-authors developing the algorithm were blinded to all data collected during the randomized session until the end of the statistical comparisons.

## 4. Sensor Fusion/Preprocessing

In order to identify specific recorded actions, current research recommends using classification features that are tailored to the application in order to best discriminate the motion gestures [19]. In a body motion tracking problem, it is therefore promising (and intuitive) to develop a pose estimator to first process the raw data (rotation rates, acceleration, magnetic field) and produce the orientation angles in inertial space to incorporate the arm elevation relative to the ground as a feature, among others. Angular rate measurements (given by rate gyroscopes) can be used to improve a tracker’s sensitivity to low rotation rates and provide an improved estimate of the device’s orientation, which was a problem in some previous work that used accelerometers only instead of a full IMU.

**Figure 3:**
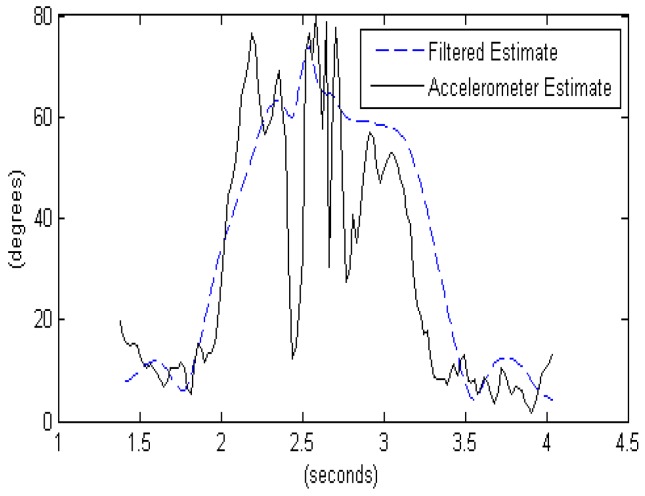
Accelerometer-only orientation estimates compared to AHRS algorithm used for a throwing motion.

Integrating angular rates is an effective way to track orientation changes with high accuracy. However, estimate drift occurs as a result of sensor bias and noise through the integration process. Utilizing direct measurements of the “nadir” vector (Earth gravity vector measured by the accelerometer triplet), and the magnetic North (magnetometer triplet), the drift can by extracted by periodic updates. In this work we use such an Attitude and Heading Reference System (AHRS) as a pre-processing step [29]. Figure 3 shows how the AHRS algorithm can improve the orientation estimates compared to using only accelerometers that couple the 1 g gravity vector with linear acceleration the device is experiencing, making orientation estimates relative to the gravity vector especially problematic for high intensity actions such as a baseball throw. An AHRS sensor fusion algorithm utilizes knowledge of the dynamics to propagate the orientation changes based on the rate gyroscope data and fuses the propagated estimate with the direct orientation estimate based on the accelerometer and compass. This way, the AHRS can tolerate shocks and vibration and maintain a stable estimate of the device’s orientation.

The accelerometer estimate of arm elevation in Figure 3 is found by comparing the acceleration along the *x*-axis to the total vector magnitude:

$$\theta_{Accelerometer\;Only}=\;\cos^{-1}\left(\frac{a_{x}}{∥\vec{a}∥}\right)$$

where $a_{x}$ is the acceleration along the *x*-axis, which points along the arm, and $∥\vec{a}∥$ is the vector magnitude of the sensed acceleration.

The filtered estimate of arm elevation in Figure 3 utilizes the attitude (orientation) estimate produced by the AHRS algorithm and finds the angle between the *z*-axis of the world reference frame and the *x*-axis of the device, as follows:

$$\theta =\;\cos^{-1}\left(\vec{u_{x}}\;\cdot \;\vec{-v_{z}}\right)$$

$$\theta_{Filtered}=\;\cos^{-1}\left(\left(DCM\times \;\left[\begin{array}{c} 1\\ 0\\ 0 \end{array}\right]\right)\;\cdot \;\left[\begin{array}{c} 0\\ 0\\ -1 \end{array}\right]\right)$$

where $-\vec{v_{z}}$ is the negative *z*-axis in the world reference frame (pointing nadir, or straight down), which is represented as [0 0 −1]^T^. $\vec{u_{x}}$ is the *x*-axis of the device (pointing along the arm), which is rotated to the world reference frame using the Direction Cosine Matrix (DCM), a rotation matrix that defines the orientation of the device relative to the world reference frame. The rotated value is represented by the matrix multiplication DCM × [100]^T^. The inverse cosine of the dot product of the two vectors results in the angle between the two unit vectors.

## 5. Data Analysis

The collected data was processed to produce a replay of the arm’s orientation as a function of time. Also, relatively simple histograms and statistics were found to provide valuable information regarding the number of times the arm moved at a high angular rate or exceeded a threshold angle. For example, patients following glenoid labral repair are often instructed not to lift their arm above their shoulder (above 90°) to allow tissues to heal for a period of time [30]. This can be easily monitored and counted using our approach.

**Figure 4:**
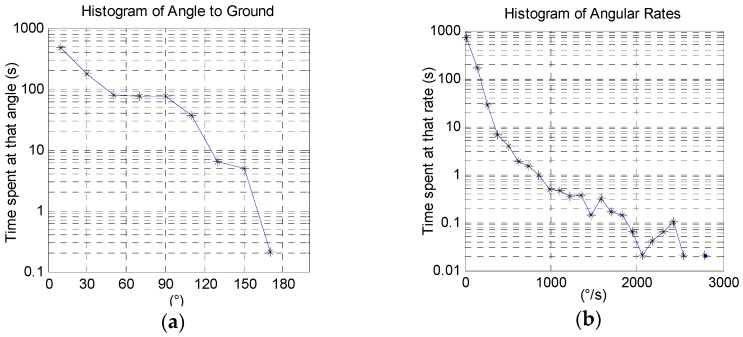
(**a**) Histogram of the angle between the device *x*-axis and nadir; (**b**) histogram of angular rate magnitude. The data are from a 15.5 min recording of a subject performing the exercises in Table 1.

Figure 4a shows a histogram for an approximately 15-min long recording of a healthy subject performing the eight repetitions of each exercise outlined in Table 1. In the figure, we observe that the arm spent the majority of the time at low angles resting. Figure 4b similarly shows a histogram of the arm’s angular rate. These histograms show the total time spent at a certain angle or angular rate and can be useful in getting a general sense of volume of motion recorded. However, it is not clear how many times any event took place, only a total time spent at various arm states. It would be more clinically relevant to produce more distinct counts, as we present next.

Instead of generating histograms for the total number of samples at a certain condition (such as a high elevation angle or high rate), it is informative to extract the number of separate times each event took place. With a sampling rate of 50 Hz, every time the arm extends for an overhead reach, for example, numerous samples will be captured for the singular event. As this is an atypical form for a histogram, we developed our own script to produce it. The algorithm is shown in Algorithm 1. In essence, a pause of one second is required before a second “event” is counted. This will allow us to measure how many separate times the arm was at a certain elevation or rotation rate.
**Algorithm 1.** Algorithm to compute a histogram, and our modified histogram; the event counter.Hist-And-Count(*data, bin_centers, sampling_frequency*)  // Initialize (histogram, event_count, and last_occurance) arrays to the length of bin_centers.  *histogram.length = event_count.length = last_occurance.length = bin_centers.length*  // Count occurrences within 1 second as one.  *event_spread = 1 × sampling_frequency*  **for**
*i* = 1 **to**
*data.length*   *distances.length = bin_centers.length*  // Compute absolute distance between current sample and all bin center points.  **for**
*j* = 1 **to**
*bin_centers.length*  *distances*[*j*] = Absolute-Value(*bin_centers*[*j*] − *data*[*i*])  // Sort distances to find nearest bin. IX[1] is the array index of that bin.  *IX* = Get-Sorted-Index-Array(*distances*)  *Bin = IX*[*1*]  // Increment Histogram bin the data point belongs to. Classic histogram.  *histogram*[*bin*] = *histogram*[*bin*] + 1  **if**
*(i – last_occurance*[*bin*]*) > event_spread*   // Increment Even Counter bin the data point belongs to.   // only if this has not occurred in the last second.   *event_count*[*bin*] = *event_count*[*bin*] + 1  *last_occurance* [*bin*] = *i***return**
*histogram, event_count*

**Figure 5:**
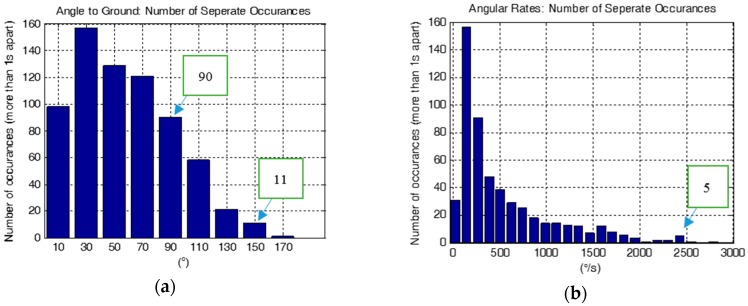
(**a**) Modified histogram of the angle between the device *x*-axis and nadir of the same data recording from Figure 4. The number of occurrences is counted, i.e., when the arm is raised to a certain angle, and only one occurrence is counted even though multiple data points recorded the event. Events must be more than one second apart to count as separate occurrences; (**b**) Histogram of angular rate magnitude. The number of occurrences is counted, i.e., when the arm rotates at a certain rate, and only one occurrence is counted even though multiple data points recorded the event. Events must be more than one second apart to count as separate occurrences.

Figure 5 shows the result of applying our event-histogram function. We observe that the arm extended to an angle between 80° and 100° a total of 90 times that are separated by more than one second, and to an angle greater than 149° a total of 11 times. We note that a motion that reaches 170° will first pass through all the lower angles, resulting in some duplicate counts. For the same data recording, Figure 5b shows a histogram of angular rate magnitude, which can be used to count high rate activities. For example, this subject reached a rate of about 2400°/s a total of five times.

## 6. Segmentation and Classification

Common classifier implementations can perform well in distinguishing recorded actions. However, two problems remain: windowing (or motion gesture spotting) [31], and the null class [32]. Windowing is non-trivial, specifically the problem of identifying when a gesture began and when it ended in the data stream. It was especially challenging when attempting to distinguish sport activities, which are typically asynchronous and high-intensity (and easier to window), from rehabilitation exercises, which include several repetitions at a time at a low rate. Automatic windowing will have false detections requiring a large classifier null-class. For example, in the simplest case, a sliding window can be used where the classifier will be engaged periodically without regard to the sensor output. For a sliding window that is one second wide and a 10 min recording, 600 data segments will be classified. We found that this greatly increases the false classification counts, even for a classifier with a low misclassification rate. When only a few seconds in total are of interest, the majority of the data segments will fall under a “null class” (an activity not of interest), which is a catch-all class that contains a wide range of actions (or inactions) that have very little in common, making it very impractical as an actual class in the classifier. We address the windowing and null class problems using a two-stage approach and an estimate of posterior probability, as described next.

## 7. Sports Activity Detection and Classification

**Figure 6:**
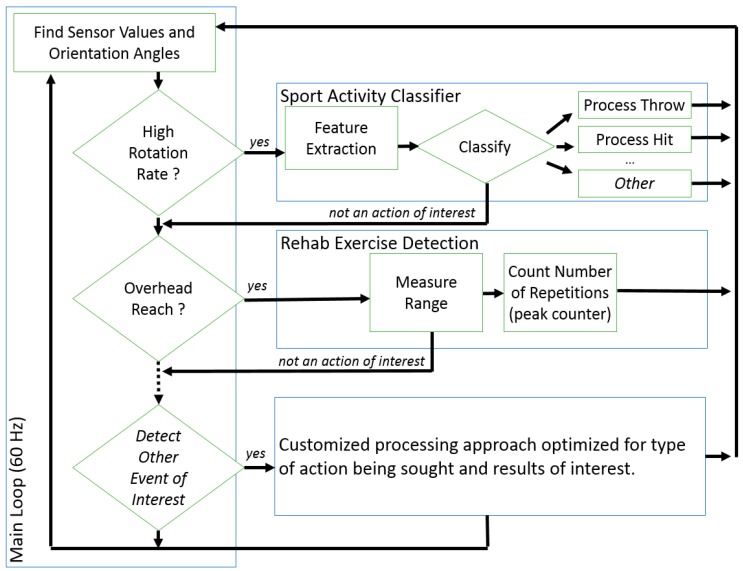
Illustration of a two-stage approach to gesture spotting and classification in real time.

Our end goal was to develop a framework that can be applied to a set of activities of any type. This can be implemented using a parallel bank of detectors and classifiers, as highlighted in Figure 6. The first stage is intended to spot a motion gesture of interest (windowing) and is a simple logical statement using the raw sensor values and orientation angles as input. This two-stage approach allows us to process the IMU data for a heterogeneous set of movements.

In this paper we demonstrate this approach for sports activities, in essence detecting and discriminating baseball throws and volleyball serves from data recordings that contain a wide range of other activities. Given that the movements of interest both involve arm elevation and a high rotation rate, we set the following first-stage rule to identify when a sport action of interest occurred reliably:

if ((Elevation of Arm > 45°) AND

(Angular Rate > 400°/s))

**Figure 7:**
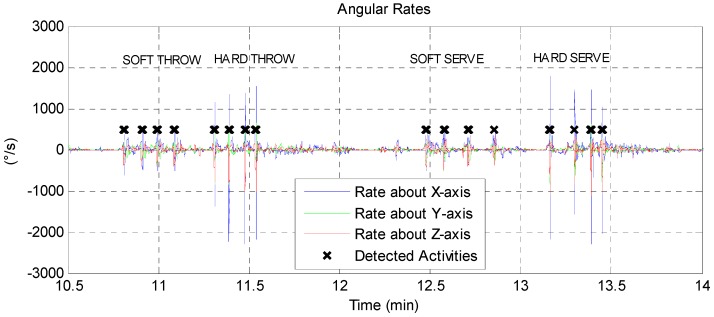
First stage detector for sport activities applied to a data segment (angular rates shown here). The segment contains 8 repetitions of baseball throws and volleyball hits (at varying intensities). *X*-marks indicate the condition: ((Elevation of Arm > 45°) AND (Angular Rate Magnitude > 400°/s)).

The results of this rule are indicated in Figure 7 for a three-minute segment. This approach rejects other occurrences of arm elevations above 45° at a lower rate (such as the Flexion exercise in Table 1). Also, moving about at a high rate would not trigger a detection unless the arm was extended. A window of fixed size is used around the detection point to extract a data segment to pass along to the second stage. The second stage, in the case of the sport activity detector, consisted of a decision tree classifier optimized for the high-rates motion-gestures. The use of the first stage detector was found to significantly reduce classifier complexity over classifier designs that included rehabilitation exercises.

Processing data from eleven subjects, a bootstrap aggregated decision tree ensemble with 160 trees was trained, using 40% of the data samples, to identify throws and serves using extracted features as described previously. Cross validation was done on the remaining 60% of the data points that served as a testing set. The feature vector consisted of 81 values for each data segment, including the minimum, maximum, range, and median value of the acceleration, rotation rate, and elevation relative to the gravity vector, for all three orthogonal axes, for the extracted sample. Features also included the rotation rate at the maximum elevation angle, the rotation axis at the maximum rotation rate, as well as values of the Fast Fourier Transform (FFT) of the rotation rate about each axis. Forward sequential feature selection was used to reduce the classifier to use eight parameters. In forward sequential feature selection, features are sequentially added to the model until there is no more improvement in prediction. Selected features are the *X* and *Z* components of the rotation axis at the point of maximum rotation rate, the range of motion of the *X*-axis relative to gravity. The remaining features are the mean FFT values of the rotation rate as follows: between 5–10 Hz and 10–25 Hz around the *Y*-axis, between 0–0.5 Hz and 2.5–5 Hz around the *Z*-axis, and between 0.5–1.5 Hz around the *X*-axis. The relatively large number of parameters helps address the null-class challenge by recognizing gestures that are neither throws nor serves.

Regarding the null-class problem, we use the classifier class scores to measure the level of fit of the data segment to the class it has been assigned to. A motion gesture must fit either the throwing or serving class with at least 60% probability; otherwise, it is considered not to belong to either class.

Table 2 shows the confusion matrix when the sports action detector and the classifier is applied to the entire dataset of eleven subjects performing multiple repetitions of the activities outlined in Table 1. The accuracy is 94.04% (sum of diagonal elements divided by overall sum). We noted the low count of “Neither” class events, where non-sport actions were mostly rejected by the first stage detector (trigger on high rates with arm elevation) and did not burden the classifier.

## 8. Validation

The results from the algorithm from the random set were compared to the actual number of baseball throws and volleyball hits observed using a contingency table to determine the ability of the algorithm to discern and count repetitions of the two sport activities from the random data collection set. Two Bland-Altman plots were created to describe agreement of the gold standard, visual observation, with the algorithm methods of estimated counts for throws and volleyball serves separately. Finally, a separate linear correlation coefficient was calculated between the counts from the algorithm and the observed counts for throwing and hitting separately.

The algorithm correctly counted 93/99 (93.9%) throws alone with the validated dataset. The algorithm correctly counted 86/103 (83.5%) hits alone (Table 3). The algorithm correctly identified and counted the two functional overhead motions over 86.23% of the time in the presence of other similar activities.

**Figure 8:**
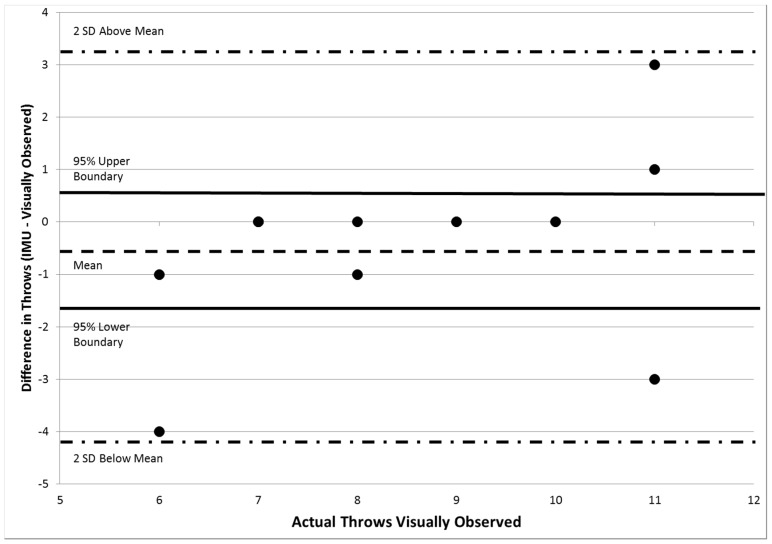
Bland-Altman Plot comparing observed and detected randomized throwing counts.

**Figure 9:**
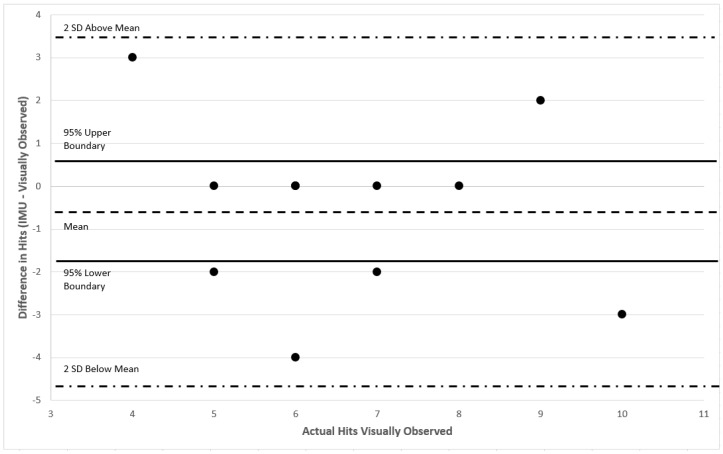
Bland-Altman Plot comparing observed and detected randomized hitting (volleyball serve) counts.

To determine the level of agreement and average error, two separate Bland-Altman plots were constructed. The average difference between the algorithm and observed counts for throwing was −0.45 (95% confidence interval −1.6:0.65) (Figure 8). The average difference between the algorithm and observed counts for volleyball hits was −0.55 (95% confidence interval −1.7:0.68) (Figure 9).

## 9. Discussion

The objective of this study was to evaluate the capabilities of an IMU with AHRS sensor fusion and a second-stage classifier to detect and discriminate between two overhead motions commonly associated with overuse injuries, throwing a baseball and serving a volleyball. The algorithm development and testing was done in a realistic scenario, where we worked with subjects with varying levels of overhead throwing experience in order to create a system that would have wide application to all skill levels from beginner to professional. Eleven random activities performed by eleven different subjects yielded 121 total events of recorded data. Sixty-three percent of the data acted as the null class, posing a significant challenge for detector and classifier implementations. The shoulder exercises, representing the null class, included several overhead motions that resemble overhead throwing and hitting that had to be ruled out as throws or volleyball hits [25].

We investigated the capability of accurately counting overhead repetitive motions from a blinded randomized data collection session. This would simulate a real-life environment in which individuals would be doing a variety of upper extremity tasks beyond repetitively overhead motions. The results support our hypothesis that the presented algorithm can differentiate motions with moderate accuracy. These findings suggest that a single IMU device applied to the arm can, with the appropriate algorithm, recognize specific movement patterns and count overhead arm volume with reasonable accuracy, without direct supervision. The capability of repetition quantification will allow coaches and healthcare providers to understand the total demand placed on the upper extremity during sport performance. It is clear that increases in throwing volume have been closely correlated with increases in injury risk to the shoulder and elbow [33,34,35,36,37,38].

The average differences between the estimated outputs and the observed counts of −0.45 for throwing a baseball and −0.55 for hitting a volleyball were very low. A difference of 0 indicates complete agreement between the algorithm and observed repetition counts. A negative average difference demonstrates a slight underestimation of the number of repetitions within a testing session. The overall accuracy was 86% in the validation data set. This compares favorably with a recent study investigating the validity of step counters [39,40]. Taking into consideration the complexity of overhead motions moving through large arcs of motion three-dimensionally, the relative accuracy of the algorithm is very good. Furthermore, the step counters were only counting steps, but our approach could differentiate two relatively similar overhead motions [41,42]. Future improvements to the classifier by collecting data with professional athletes can improve accuracy, essentially by allowing the classifier to have narrower definitions of a throw or serve.

Data acquisition occurred in a clinical laboratory setting, and the sample size for this design was relatively small with varying levels of experience. Testing a larger sample size will allow more confidence in concluding the true accuracy of this method of activity volume quantification. In addition, we expect a sample with a consistent level of competitive participation status will increase the accuracy of the device and algorithm, due to decreased variability in task biomechanics and velocity. Using the device in the field setting with specific athletic population will more likely provide an accurate representation of the system’s validity as the number of throws will increase, providing a more accurate representation of an error rate.

## 10. Conclusions

A sports activity tracker was developed using IMU data recordings from eleven subjects performing overhead exercises, serving a volleyball and throwing a baseball. The first stage detector was designed to trigger at instances where the arm was elevated and moving at a high rotation rate. This worked well at excluding all other activities recorded by the IMU. As the second stage, a decision tree classifier was implemented to identify whether the motion was a baseball throw, a volleyball hit, or neither. Training, testing, and randomized validation data with blinding were collected. With 86% accuracy, the described algorithm was able to count the number of throws and hits the subjects had performed in the validation dataset, which included a large number of other similar activities that serve as a realistic “null class.” This established the capability of tracking arm motions with moderate accuracy to provide coaches and medical staff with reasonable estimates of throwing volumes, which have been linked to overuse injuries of the upper extremity.

## Figures and Tables

**Table 1:** Outline of performed activities under IRB #13-0602-F1V at the University of Kentucky.

Category	Sub-Category	Action	Description/Subject Instructions
Sport Activities	Overhead	Baseball Throw	Throw baseball at best effort
		Volleyball Serve	Hit the volleyball serve at best effort
Shoulder Exercises	Throwing Plane of Motion	External Rotation at 90	Raise arm so your elbow is level with your shoulder, then rotate your hand back in line with your ear
		Throw Deceleration	From the arm cocking position go through the throwing motion slowly. Resistance is in front of the person
		Throw Acceleration	From the arm cock position go through the throwing motion slowly. Resistance is at the rear of the person
	Sagittal Plane	Flexion	Lift your arm up overhead
		Extension	Pull your arm down next to your body
	Other	External Rotation at Side	Rotate your arm out to the side with your elbow at your side like you are opening a door
		Scapular Punch	Reach forward against the resistance of the band with elbow in full extension; now punch your arm forward and back without bending your elbow

**Table 2:** Confusion matrix for the sport activity classifier over training and testing datasets.

		Predicted Class		
		Throw	Serve	Neither
*True Class*	Throw	131	3	0
	Serve	2	134	3
	Neither	5	4	3

**Table 3:** Confusion matrix for the sport activity classifier over validation dataset (randomized counts and order, and blinded).

		Predicted Class		
		Throw	Serve	Neither
*True Class*	Throw	93	6	0
	Serve	4	86	3
	Neither	18	8	4
